# Predictive Value of Fasting Blood Glucose for Microvascular Obstruction in Nondiabetic Patients with ST-Segment Elevation Myocardial Infarction after Primary Percutaneous Coronary Intervention

**DOI:** 10.1155/2020/8429218

**Published:** 2020-09-26

**Authors:** Han Wu, Ran Li, Kun Wang, Dan Mu, Jian-Zhou Chen, Xuan Wei, Xue Bao, Zhong-Hai Wei, Jun Xie, Biao Xu

**Affiliations:** ^1^Department of Cardiology, Drum Tower Hospital, Medical School of Nanjing University, Nanjing, China; ^2^Department of Radiology, Drum Tower Hospital, Medical School of Nanjing University, Nanjing, China; ^3^Department of Cardiology, Nanjing Drum Tower Hospital Clinical College of Nanjing Medical University, Nanjing, China

## Abstract

**Background:**

The relationship between fasting blood glucose (FBG) and microvascular obstruction (MVO) after primary percutaneous coronary intervention (PCI) remains unclear in nondiabetic patients with ST-segment elevation myocardial infarction (STEMI). This study aimed to determine the predictive value of FBG in MVO in nondiabetic STEMI patients.

**Methods:**

A total of 108 nondiabetic STEMI patients undergoing primary PCI were enrolled in this study. The patients were classified into either the MVO group or non-MVO group based on cardiac magnetic resonance imaging (CMR).

**Results:**

FBG in the MVO group was higher than in the non-MVO group. Univariate analysis showed that FBG, peak high-sensitive troponin T (TnT), pre-PCI thrombolysis in myocardial infarction (pre-PCI TIMI) flow, left ventricular ejection fraction (LVEF), infarction size, left ventricular end-diastolic diameter (LVEDd), left ventricular end-diastolic volume (LVEDV), and global longitudinal strain (GLS) were likely predictive factors of MVO. After adjustment for other parameters, FBG, peak TnT, LVEF, and LVEDV remained independent predictors for MVO.

**Conclusion:**

FBG was independently associated with MVO in nondiabetic STEMI patients.

## 1. Introduction

Timely reperfusion therapy with primary percutaneous coronary intervention (PCI) has been the preferred treatment for most ST-segment elevation myocardial infarction (STEMI). However, a sizeable proportion of patients exhibited impaired microvascular reperfusion despite epicardial coronary vessel patency, defined by microvascular obstruction (MVO) [1]. In the past few years, much attention has been devoted to assess the role of MVO in acute myocardial infarction. Pooled data from seven randomized trials showed that MVO detected by cardiac magnetic resonance imaging (CMR) after primary PCI was strongly associated with all-cause mortality and hospitalization for heart failure within one year [2]. A recent report demonstrated that late MVO extent increased long-term risk of major adverse events [3].

It was widely accepted that diabetes was an important risk factor for myocardial infarction. Furthermore, stress hyperglycemia was associated with left ventricular remodeling and inhospital mortality in myocardial infarction patients with anterior myocardial infarction [4]. A recent report showed that hyperglycemia on admission predicted no-reflow in STEMI patients undergoing PCI [5]. The previous studies focused on stress hyperglycemia in myocardial infarction; however, relatively little information was available about fasting blood glucose (FBG) in myocardial infarction.

Thus, the present study aimed to evaluate the predictive value of FBG on MVO in nondiabetic STEMI patients treated with primary PCI.

## 2. Methods

### 2.1. Study Patients

This retrospective observational case-control study enrolled STEMI patients who underwent primary PCI at Nanjing Drum Tower Hospital affiliated to Nanjing University Medical School between July 2018 and August 2019. The following inclusion criteria were applied: (1) patients aged 18–85 years; (2) patients diagnosed with STEMI according to the newest guideline [6]; (3) patients who had indication for primary PCI; and (4) patients agreed to accept CMR examination. Exclusion criteria included the following: (1) previous diabetes; (2) old myocardial infarction; (3) severe inflammatory diseases; (4) any type of malignancies; (5) severe hepatic or renal diseases; (6) thrombolysis before PCI; and (7) magnetic resonance imaging (MRI) contraindication. The study protocol was approved by the research ethics committee of Drum Tower Hospital affiliated to Nanjing University Medical School. Finally, 108 participants were divided into either MVO or non-MVO group according to the level of MVO detected by CMR within 7 days after primary PCI.

### 2.2. Primary PCI

All the enrolled patients received dual antiplatelet therapy (a loading dose of aspirin 300 mg and 600 mg clopidogrel/180 mg ticagrelor) before primary PCI. The procedures were performed in line with the current recommended guidelines. The weight-adjusted dose of heparin (100 U/kg without glycoprotein IIb/IIIa inhibitor or 70 U/kg with glycoprotein IIb/IIIa inhibitor) was administered throughout the procedure. Manual thrombus aspiration, stent length, stent diameter, and glycoprotein IIb/IIIa inhibitor (tirofiban) were left to the operator's discretion. Angiographic findings including infarct-related artery (IRA), pre-PCI thrombolysis in myocardial infarction (pre-PCI TIMI), thrombus aspiration, syntax score, stent length, stent diameter, tirofiban, and post-PCI TIMI were collected.

### 2.3. Demographic Data, Laboratory Assays, and Echocardiography

Demographic characteristics (age, gender, heart rate, body mass index, Killip class, blood pressure, and symptom onset to balloon time) and previous history (preinfarction angina, smoking, and hypertension) were recorded in medical files while hospitalized. Fasting blood samples were collected on the next day after admission to measure creatinine, C-reactive protein (CRP), total cholesterol, high-density lipoprotein (HDL), low-density lipoprotein (LDL), FBG, and hemoglobin A1c (HbA1c). Blood samples were drawn from patients for brain-type natriuretic peptide (BNP) detection before primary PCI. However, high-sensitive troponin T (TnT) levels were detected on admission, 6 hours after primary PCI, and every day up to 3 days to identify peak values.

Two-dimensional transthoracic echocardiography was performed within 2 days after primary PCI. Left ventricular ejection fraction (LVEF) was evaluated using Simpson's biplane technique. Left atrial diameter (LAD) and left ventricular end-diastolic diameter (LVEDd) were obtained by echocardiography.

### 2.4. CMR

CMR protocol and standardized postprocessing were conducted on 3.0 T CMR for left ventricular function and standard infarction characteristics (infarct size, MVO, and strain) as previously described [3]. Briefly, cine images were obtained 5 minutes after injection of extracellular gadolinium-based contrast agent for left ventricular function. Endocardial and epicardial borders were manually traced to calculate left ventricular end-diastolic volume (LVEDV). MVO was identified as a region of the hypointense area in the score of late gadolinium enhancement images and was quantified as a percentage of the left ventricle. Infarction size was measured by delayed enhancement CMR images and expressed as percentage of left ventricle mass. The global radial, circumferential, and longitudinal strains were detected as previously described [7].

### 2.5. Statistical Analysis

Continuous variables were expressed as means ± standard deviation (normal distribution) or median with interquartile range (nonnormal distribution). Categorical data were depicted as numbers (*n*) with percentages (%). Differences between continuous variables were analyzed using Student's *t*-test (normal distribution) or Mann–Whiney's *U* test (nonnormal distribution). Categorical variables were compared by the chi-square test. However, univariate regression analysis was used to determine the available variables predicting MVO in STEMI patients undergoing primary PCI. Any variable with unadjusted *p* < 0.1 was included in the multivariate logistic regression analysis. Spearman correlation coefficient was used to test the relationships between continuous variables. The receiver-operating characteristic (ROC) curve was performed to determine the optimum cutoff value and the sensitivity and specificity of FBG to predict MVO. Statistical analyses were performed with R and EmpowerStats software.

## 3. Results

### 3.1. Basic Clinical and Laboratory Characteristics of Patients

**Figure 1:**
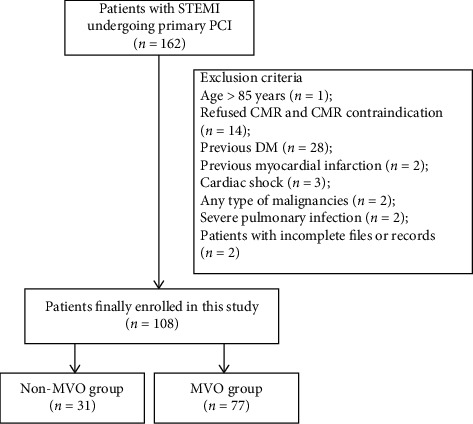
Flowchart of the study selection process. STEMI, ST-segment elevation myocardial infarction; PCI, percutaneous intervention; CMR, cardiac magnetic resonance; DM, diabetes mellitus; MVO, microvascular obstruction.

**Figure 2:**
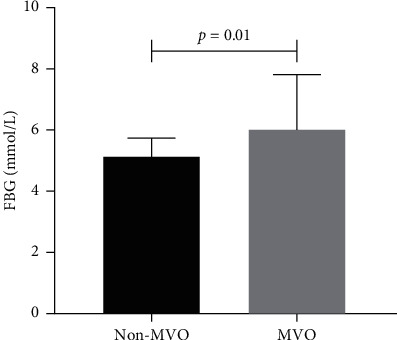
Fasting blood glucose in non-microvascular obstruction and microvascular obstruction groups. FBG, fasting blood glucose; MVO, microvascular obstruction.

Initially, 162 patients were eligible for this research, and 54 patients were excluded. Therefore, 108 patients were finally included in our analysis (Figure 1). The demographic characteristics, baseline information, and hematological parameters of the groups were presented in Table 1. No significant differences were observed between the two groups with respect to age, gender, hypertension, preinfarction angina, smoking, body mass index, blood pressure, heart rate, Killip class, and symptom onset to balloon time (*p* > 0.05 for all). The comparison of laboratory parameters of the two groups showed no statistically significant differences apart from FBG and peak TnT. The patients in the MVO group had significantly higher FBG levels compared to the non-MVO group (6.0 ± 1.8 vs 5.2 ± 0.6, *p*=0.01, Table 1 and Figure 2). Besides, peak TnT was higher in the MVO group (6.7 (4.2–10.0) vs 2.6 (1.5–3.7), *p* < 0.001).

### 3.2. Procedural, Echocardiographic, and CMR Characteristics of Patients

Table 2 summarizes the interventional, echocardiographic, and CMR data of the two groups. No significant differences were found in terms of interventional characteristics except for pre-PCI TIMI flow. Pre-PCI TIMI grade 3 was less frequent in the MVO group than in the non-MVO group (3.9% vs 16.1%, *p*=0.042). For echocardiographic parameters, LVEF was lower among patients with MVO than those without (44.9 ± 4.6 vs 49.5 ± 4.5, *p* < 0.001). LVEDd in the MVO group was larger compared to the non-MVO group (5.5 ± 0.4 vs 5.3 ± 0.3, *p*=0.016). Although the average diameters of the left atrium in MVO subgroup participants were higher than that of the non-MVO subgroup, it did not reach a significant difference (4.1 ± 0.4 vs 4.0 ± 0.3, *p*=0.076). With respect to CMR parameters, patients with MVO showed larger infarction size (21.0 (17.0–30.2) vs 15.2 (9.0–21.0), *p*=0.007) and higher LVEDV (93.5 ± 29.9 vs 70.9 ± 21.8, *p* < 0.001). Furthermore, MVO was significantly associated with the global circumferential strain (GCS) (*p*=0.001).

### 3.3. Predictors of MVO

As shown in Table 3, univariate regression analysis revealed that FBG, peak TnT, pre-PCI TIMI = 3, LVEDd, LVEF, infarction size, LVEDV, and GLS were associated with MVO. Multivariate analysis showed that FBG (OR, 2.49; 95% CI, 1.13–5.47; *p*=0.0237), peak TnT (OR, 1.27; 95% CI, 1.02–1.59; *p*=0.0322), LVEF (OR, 0.77; 95% CI, 0.63–0.94; *p*=0.0112), and LVEDV (OR, 1.04; 95% CI, 1.01–1.07; *p*=0.0223) remained independent predictors for the development of MVO in nondiabetic STEMI patients undergoing primary PCI.

## 4. The ROC Curve of FBG for Predicting MVO

**Figure 3:**
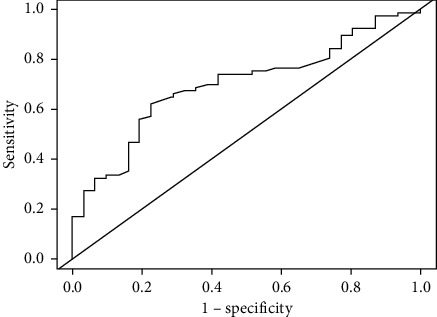
Receiver-operating characteristic curve of predicting the microvascular obstruction phenomenon after primary percutaneous intervention of nondiabetic ST-segment elevation myocardial infarction patients.

ROC curve analysis was performed to detect the effect of FBG in the prediction of MVO development (Figure 3). The ROC area of FBG was 0.70 (95% CI: 0.59–0.80), and FBG above 5.47 mmol/L had 62.3% sensitivity and 77.4% specificity for predicting MVO.

## 5. Discussion

The present study is the first, to our knowledge, to explore the association between FBG and MVO in nondiabetic STEMI patients undergoing primary PCI. As shown in the study, MVO was very common in STEMI patients undergoing primary PCI, and CMR was an effective and safe method to detect MVO in STEMI patients within one week after primary PCI. Furthermore, peak TnT, LVEF, and LVEDV were independent predictive factors of MVO in STEMI. In addition, we demonstrated that patients with MVO exhibited higher FBG than those without, and FBG was independently associated with MVO.

In the reperfusion era, primary PCI was the preferred reperfusion treatment in STEMI patients, improving clinical prognosis [8]. Despite successful mechanical opening of IRA, impaired myocardial and microvascular reperfusion frequently occurred in the patients after primary PCI, which was defined by MVO [1]. The presence and extent of MVO after primary PCI was independently associated with long-term mortality and hospitalization for heart failure in STEMI patients [2, 3, 9]. Several techniques such as TIMI flow, myocardial blush grade (MBG), electrocardiogram, CMR, single-photon emission computed tomography, and positron emission tomography were used to diagnose MVO. However, late gadolinium enhancement CMR was the current cutting edge method for diagnosis and quantification of MVO [8]. Thus, in the present study, CMR was used to detect MVO in nondiabetic STEMI patients after primary PCI.

Growing evidence has illustrated that high glucose was associated with clinical outcomes of STEMI with PCI [10, 11]. In addition, increased admission glucose impaired coronary flow in STEMI patients [12, 13]. To the best of our knowledge, this was the first study showing that FBG was independently associated with CMR-measured MVO after primary PCI in nondiabetic STEMI patients. Supporting this statement, a recent study detected microvascular function using alteration of resting flow and baseline microvascular resistance and showed that admission glucose contributed to microvascular dysfunction in nondiabetic anterior-wall STEMI patients [14]. Furthermore, acute hyperglycemia attenuated the protective effect of preinfarction angina on microvascular function after primary PCI in STEMI patients [15]. These studies collectively suggested that high FBG promoted MVO in STEMI.

Though the association between FBG and MVO was established in this study, the underlying mechanisms were not fully understood. As discussed in a previous review, individual susceptibility, ischemia-related injury, reperfusion-related injury, and distal embolization were the four interacting mechanisms involved in coronary MVO [1]. Firstly, patients with diabetes and acute hyperglycemia were susceptible to MVO in myocardial ischemia [1]. Secondly, stress high glucose significantly induced apoptosis of cardiac microvascular endothelial cells in cell experiments [16, 17]. Furthermore, several experimental and clinical studies have showed that high glucose promoted myocardial cell injury [11, 18], which may lead to myocardial cell swelling during ischemia-related injury. Thirdly, high glucose challenge induced oxidative stress and inflammatory cytokines in cardiac cells [19]. In addition, high glucose induced endothelin-1 and inflammation in endothelial cells [20], suggesting that high glucose might promote microvascular vasoconstriction. These studies showed that high glucose could exaggerate reperfusion-related injury. Finally, numerous studies have demonstrated that high glucose enhanced platelet activation and thrombin responses [21, 22]. Thus, high glucose-induced thrombus might contribute to distal embolization during MVO.

Patients with MVO exhibited significantly higher peak TnT, peak CK-MB, and infarction size, which was consistent with the results of others [23–25]. Consistent with the clinical observations demonstrating an association between MVO and LVEF [25–27], we found that LVEF in the MVO group was significantly lower than that of the non-MVO group. Wong and colleagues indicated that CMR-derived assessment of MVO was a strong predictor of left ventricular dysfunction at three months follow-up [28]. Recently, another study showed an association between non-IRA MVO and worse persistent diastolic dysfunction in revascularized STEMI patients [29]. In line with these studies, the present study suggested that LVEF was independently associated with occurrence of MVO after adjustment of other parameters.

Several studies have illustrated that longitudinal strain was significantly correlated with MVO development in infarcted myocardium of patients with STEMI [30, 31]. Additionally, circumferential strain predicted the recovery of LV systolic function and late myocardial remodeling in myocardial infarction [32, 33]. However, in the present study, GCS was not a predictor for MVO after adjustment of other parameters.

## 6. Conclusion

Our findings indicated that FBG was independently associated with MVO in nondiabetic STEMI patients undergoing primary PCI.

## 7. Limitations

The present study is a small sample observational study, and some biases might exist in this study. Besides, the nondiabetic patients enrolled in this study referred to no history of diabetes; thus, several patients might be newly diagnosed diabetes in the participants. Finally, several parameters related to CMR were not evaluated in the study because of limited techniques.

## Figures and Tables

**Table 1:** Baseline clinical characteristics of the participants.

	Non-MVO (*N* = 31)	MVO (*N* = 77)	*P* value
Age, years	62.4 ± 11.7	60.1 ± 12.7	0.379
Male, *n* (%)	24 (77.4)	70 (90.9)	0.109
Hypertension, *n* (%)	15 (48.4)	38 (49.4)	0.928
Preinfarction angina	11 (35.5)	18 (23.4)	0.199
Smoking, *n* (%)	17 (54.8)	45 (58.4)	0.732
Body mass index, kg/m^2^	24.7 ± 2.7	24.8 ± 3.1	0.830
Systolic blood pressure, mmHg	133.4 ± 24.4	127.7 ± 25.6	0.291
Diastolic blood pressure, mmHg	83.5 ± 10.2	84.2 ± 13.1	0.788
Heart rate, beats per min	79.9 ± 14.5	78.1 ± 16.2	0.600
Killip class, *n* (%)			0.215
I	29 (93.5)	68 (88.3)	—
II	1 (3.3)	6 (7.8)	—
III	1 (3.3)	0 (0.0)	—
IV	0 (0.0)	3 (3.9)	—
Symptom onset to balloon time, hours	4.8 (3.0–8.0)	5.0 (3.5–8.0)	0.835
Creatinine, *μ*mol/L	72.5 ± 18.6	71.6 ± 14.5	0.796
CRP	4.8 (3.2–9.4)	6.3 (3.7–14.8)	0.181
Total cholesterol, mmol/L	4.2 ± 0.8	4.1 ± 0.8	0.797
HDL, mmol/L	1.0 ± 0.3	1.0 ± 0.3	0.894
LDL, mmol/L	2.5 ± 0.7	2.5 ± 0.7	0.959
FBG, mmol/L	5.2 ± 0.6	6.0 ± 1.8	0.010
HbA1c, %	5.8 ± 0.5	6.0 ± 1.1	0.264
Peak TnT, *μ*g/L	2.6 (1.5–3.7)	6.7 (4.2–10.0)	<0.001
BNP, pg/mL	33.1 (19.0–104.0)	65.9 (24.1–209.8)	0.356

MVO, microvascular obstruction; CRP, C-reactive protein; HDL, high-density lipoprotein; LDL, low-density lipoprotein; FBG, fasting blood glucose; HbA1c, hemoglobin A1c; TnT, troponin T; BNP, B-type natriuretic peptide.

**Table 2:** Procedural, echocardiographic, and CMR parameters of the study cohort.

		Non-MVO (*N* = 31)	MVO (*N* = 77)	*P* value
IRA, *n* (%)		—	—	0.229
Left anterior descending artery		13 (41.9)	45 (58.4)	—
Left circumflex artery		4 (12.9)	10 (13.0)	—
Right coronary artery		14 (45.2)	22 (28.6)	—
Thrombus aspiration, *n* (%)		10 (32.3)	33 (42.9)	0.309
Syntax score		15.5 (9.0–22.2)	15.0 (11.0–22.5)	0.736
Stent length, mm		33.0 (20.5–38.0)	33.0 (23.0–46.0)	0.333
Stent diameter, mm		2.8 ± 1.2	3.0 ± 0.8	0.173
Use of tirofiban, *n* (%)		20 (64.5)	53 (68.8)	0.665
Pre-PCI TIMI = 3, *n* (%)		5 (16.1)	3 (3.9)	0.042
Post-PCI TIMI = 3, *n* (%)		31 (100.0)	74 (96.1)	0.556
LVEDd, cm		5.3 ± 0.3	5.5 ± 0.4	0.016
LAD, cm		4.0 ± 0.3	4.1 ± 0.4	0.076
LVEF, %		49.5 ± 4.5	44.9 ± 4.6	<0.001
Infarct size, % LV		15.2 (9.0–21.0)	21.0 (17.0–30.2)	0.007
MVO, % LV		0.0 (0.0–0.0)	2.0 (1.0–3.7)	<0.001
LVEDV, mL		70.9 ± 21.8	93.5 ± 29.9	<0.001
GRS, %		24.3 ± 7.9	21.1 ± 7.3	0.053
GCS, %		−16.2 ± 3.7	−13.8 ± 3.1	0.001
GLS, %		−9.9 ± 3.0	44.9 ± 4.6	0.059

MVO, microvascular obstruction; IRA, infarct-related artery; pre-PCI TIMI, pre-percutaneous intervention thrombolysis in myocardial infarction; post-PCI TIMI, post-percutaneous intervention thrombolysis in myocardial infarction; LVEDd, left ventricular end-diastolic diameter; LAD, left atrial diameter; LVEF, left ventricular ejection fraction; LVEDV, left ventricular end-diastolic volume; GRS, global radial strain; GCS, global circumferential strain; GLS, global longitudinal strain.

**Table 3:** Univariate and multivariate logistic analysis of predictors of MVO in nondiabetic STEMI patients after primary PCI.

	Univariate analysis		Multivariate analysis	
	Odds ratio (95% CI)	*P* value	Odds ratio (95% CI)	*P* value
Age	0.98 (0.95, 1.02)	0.376	—	—
Sex (female)	0.34 (0.11, 1.08)	0.067	—	—
FBG	2.47 (1.31, 4.68)	0.005	2.49 (1.13, 5.47)	0.0237
Peak TnT	1.42 (1.20, 1.66)	<0.001	1.27 (1.02, 1.59)	0.0322
Pre-PCI TIMI = 3	0.21 (0.05, 0.94)	0.042	—	—
LVEDd	4.74 (1.30, 17.33)	0.019	—	—
LVEF	0.80 (0.71, 0.89)	<0.001	0.77 (0.63, 0.94)	0.0112
Infarct size (% LV)	1.06 (1.02, 1.12)	0.010	—	—
LVEDV	1.04 (1.02, 1.06)	<0.001	1.04 (1.01, 1.07)	0.0223
GLS	1.15 (0.99, 1.33)	0.064	—	—

MVO, microvascular obstruction; STEMI, ST-segment elevation myocardial infarction; PCI, percutaneous intervention; FBG, fasting blood glucose; TnT, troponin T; pre-PCI TIMI, pre-percutaneous intervention thrombolysis in myocardial infarction; LVEDd, left ventricular end-diastolic diameter; LVEF, left ventricular ejection fraction; LVEDV, left ventricular end-diastolic volume; GLS, global longitudinal strain.

## Data Availability

The data used to support the findings of this study are available from the corresponding author upon request.
